# Clinical Bioequivalence of Wixela Inhub and Advair Diskus in Adults With Asthma

**DOI:** 10.1089/jamp.2019.1547

**Published:** 2020-04-02

**Authors:** Dik Ng, Edward M. Kerwin, Martha V. White, S. David Miller, Scott Haughie, Jonathan K. Ward, Richard Allan

**Affiliations:** ^1^ Mylan Pharma UK Ltd., Sandwich, Kent, United Kingdom.; ^2^ Clinical Research Institute of Southern Oregon, Medford, Oregon.; ^3^ Institute for Asthma and Allergy, Wheaton, Maryland.; ^4^ Northeast Medical Research Associates, Inc., North Dartmouth, Massachusetts.

**Keywords:** Advair Diskus, Wixela Inhub, fluticasone propionate, salmeterol, local bioequivalence, generic drugs

## Abstract

***Background:*** Wixela^®^ Inhub^®^ is a dry powder inhaler approved as a generic equivalent to Advair^®^ Diskus^®^ (fluticasone propionate [FP]/salmeterol fixed-dose combination) for patients with asthma or chronic obstructive pulmonary disease (COPD). This study aimed at confirming the local (lung) therapeutic equivalence of both the FP and salmeterol components of Wixela Inhub (test [T]) to Advair Diskus (reference [R]) after inhalation.

***Methods:*** This randomized, double-blind, double-dummy, placebo-controlled, parallel-group study in patients ≥18 years with mild-to-moderate persistent asthma compared the local therapeutic equivalence (using forced expiratory volume in 1 second [FEV_1_]) of FP/salmeterol (100/50 μg) after inhaled delivery via T and R.

***Results:*** Randomized patients (*N* = 1127) received T (*n* = 512), R (*n* = 512), or placebo (*n* = 103). T and R significantly increased day 1 FEV_1_ area under the effect curve over 12 hours of the change from baseline (AUC_[0–12]_) and day 29 trough FEV_1_ over placebo, indicating that these endpoints were sufficiently sensitive for evaluation of bioequivalence. On day 1, T and R each increased FEV_1_ AUC_(0–12)_ over placebo (3.134 L•h [T], 2.677 L•h [R]; each *p* < 0.0001). Following twice-daily dosing for 28 days, T and R also each increased trough FEV_1_ (measured on day 29) over placebo (235 mL [T], 215 mL [R]; each *p* < 0.0001). Least-squares mean T/R ratios (90% confidence intervals) for day 1 FEV_1_ AUC_(0–12)_ and day 29 trough FEV_1_ were 1.120 (1.016–1.237) and 1.069 (0.938–1.220), respectively, indicating that T and R were bioequivalent for both co-primary endpoints. FP/salmeterol was well tolerated when administered via either T or R.

***Conclusions:*** These results demonstrate that the therapeutic effects of Wixela Inhub are bioequivalent to Advair Diskus in the lung. Wixela Inhub represents a therapeutically equivalent new FP/salmeterol treatment option for use in the treatment of asthma and COPD.

## Introduction

Inhaled corticosteroids (ICS) and long-acting β_2_-adrenergic agonists (LABA) are widely used, safe, and effective anti-inflammatory and bronchodilator agents, respectively, for the treatment of asthma and chronic obstructive pulmonary disease (COPD).^(1,2)^ Current guidelines recommend the administration of fixed-dose ICS/LABA combination drugs as maintenance therapy in asthma and COPD.^(3–5)^ Advair Diskus (GlaxoSmithKline, Research Triangle Park, NC) is a widely prescribed ICS/LABA combination drug (fluticasone propionate [FP]/salmeterol [as xinafoate]; FPS) for asthmatic patients not controlled with ICS alone and for COPD patients at high risk of exacerbations.^(1,2)^ With the expiration of the US patent for Advair Diskus in 2016, several generic versions are currently advancing toward regulatory approval.^(6–9)^ The most advanced of these, in terms of drug development stage in the United States, is Wixela Inhub, composed of FPS inhalation powder (Mylan, Inc., Canonsburg, PA) predispensed in a multidose inhaler (Inhub; Mylan, Inc.),^(10,11)^ which was recently approved by the US Food and Drug Administration (FDA).^(12)^

The FDA guidelines for the development of generic FPS inhalers require, as part of a weight of evidence approach (together with *in vitro* pharmaceutical equivalence and systemic pharmacokinetic bioequivalence), local (lung) therapeutic equivalence studies that, in total, demonstrate therapeutic equivalence to Advair Diskus.^(13)^ Clinical development of Wixela Inhub followed these guidelines, and recent studies confirmed the pharmacokinetic bioequivalence of single doses of Wixela Inhub for each of the three authorized Advair Diskus dose strengths.^(14)^ Here, we report the results of the FDA-mandated local therapeutic equivalence study (NCT02245672) in adult patients with asthma.

The objective of this study was to compare the clinical efficacy of the FP and salmeterol components of Wixela Inhub 100/50 μg and Advair Diskus 100/50 μg by using spirometry. To evaluate bioequivalence of the bronchodilator component (salmeterol), forced expiratory volume in 1 second (FEV_1_) was measured repeatedly for 12 hours after the first study dose. The anti-inflammatory component (FP) was then evaluated by measuring trough FEV_1_ after 28 days of twice-daily dosing.

## Materials and Methods

In this article, “test product” (T) and “reference product” (R) are defined as follows: T is Wixela Inhub (FPS administered via the Inhub inhaler), and R is Advair Diskus.

### Study design and conduct

This multicenter, randomized, double-blind, double-dummy, placebo-controlled, parallel-group study was conducted between October 22, 2014 and July 10, 2015 at 101 U.S. centers. The study consisted of a 21–28-day single-blind, placebo run-in period followed by a 4-week double-blind treatment period. The primary objective was to assess the local therapeutic equivalence of T and R using spirometry.

The study conformed to appropriate ethical guidelines and was conducted in accordance with the principles of the International Conference on Harmonisation of Technical Requirements for Registration of Pharmaceuticals for Human Use guidelines for good clinical practice^(15)^ and the code of ethics of the World Medical Association's Declaration of Helsinki.^(16)^ Quorum Institutional Review Board approved the study protocol, and all patients provided written informed consent.

### Patients and treatments

Consistent with the FDA guidelines for a clinical endpoint study to assess local therapeutic equivalence of FPS products,^(13)^ key inclusion criteria included age ≥18 years with diagnosis of asthma ≥12 weeks according to National Asthma Education and Prevention Program guidelines^(3)^; a mean baseline FEV_1_ of 50%–85% predicted after ≥6 hours without short-acting bronchodilator use; postbronchodilator reversibility (percent improvement) of ≥12% within 30 minutes of 360 μg albuterol; and current nonsmokers (with no smoking history within the past 12 months and a total smoking history of ≤10 pack-years). Patients were excluded if they had a respiratory condition or another severe progressive disease other than asthma and allergic rhinitis, were hospitalized for asthma within the past year or had an asthma exacerbation within the preceding 3 months, or had a respiratory tract, sinus, or ear infection within the preceding 4 weeks.

After completion of the placebo run-in period of 21–28 days (all subjects receiving placebo for Wixela Inhub, one inhalation twice daily), eligible patients were randomly assigned to one of three groups (T, R, or placebo) in a 5:5:1 ratio by using a subject identification number assigned via an automated interactive voice-/web-response system. Each treatment was administered in a double-blind, double-dummy manner (with placebo inhalers matched to T or R used for the placebo group and to maintain the blind in the active treatment groups). Patients were required to take one inhalation twice daily from each of their assigned inhalers for 4 weeks.

Advair Diskus and Wixela Inhub contained qualitatively and quantitatively equivalent formulations of both active pharmaceutical ingredients (a fixed-dose combination of micronized crystalline FP and salmeterol [as xinafoate]) and inactive excipients (lactose monohydrate). The Diskus and Inhub inhalers were medium resistance passive dry powder inhalers, contained 60 premetered doses of FP and salmeterol, and had the same operating procedures.^(17)^

The pharmaceutical performance of multiple commercial batches of R (Advair Diskus) was tested to characterize the performance using *in vitro* methods, including measures of delivered dose and aerodynamic particle size distribution.^(18)^ The single batch of Advair Diskus used in the study was representative of the median of the Advair Diskus commercial batch population.

The T drug (Wixela Inhub) was also tested to characterize performance by using *in vitro* methods. The two batches of Wixela Inhub used in the study were manufactured at commercial scale, representative of the product in terms of *in vitro* performance, and were age-matched to be within 3 months of the batch of Advair Diskus used in the study.

The placebo for Advair Diskus used commercial stock of Advair Diskus inhalers. Specifically, these were opened under good manufacturing practice (GMP) conditions, the blister strips containing FP and salmeterol were replaced with strips containing lactose, and the inhalers were subsequently closed and packaged for clinical trial use. The placebo for Wixela Inhub used Inhub inhalers containing lactose alone.

### Assessments

Spirometry assessments were completed at screening (day −28); at run-in (day −3 to −7); at −0.5, 0, 0.5, 1, 2, 3, 4, 6, 8, 10, and 12 hours on day 1 of treatment, before dosing on day 15, and on day 29. Primary endpoints were the area under the effect curve over 12 hours (FEV_1_ AUC_(0–12)_) for the change from baseline (CFB) in FEV_1_ on day 1, the first day of treatment, and CFB in trough FEV_1_ on day 29 after 4 weeks of dosing. Safety assessments included adverse events (AEs) and laboratory safety tests, vital signs (blood pressure and pulse rate), and electrocardiograms.

### Statistical analysis

The safety set was defined as all randomly assigned patients who had taken ≥1 dose of study drug and for whom postdose safety data were available. The full analysis set (FAS) was defined as all randomly assigned patients who had taken ≥1 dose of study drug and had provided data for either co-primary efficacy endpoint (FEV_1_ AUC_(0–12)_ or day 29 CFB in trough FEV_1_). The per-protocol set (PPS) was defined as all patients in the FAS who had not violated or deviated from the protocol in a manner that could have affected the outcome of the FEV_1_ assessments for both co-primary efficacy endpoints. The FAS was the primary analysis set used to establish assay sensitivity (the ability to discriminate both T and R treatments from placebo), whereas the PPS was the primary analysis set used to establish bioequivalence between T and R treatments.

The original sample size for this study was calculated by assuming a 92% between-subject coefficient of variation (CV; expected mean and standard deviation [SD] for CFB in trough FEV_1_ on day 29 of 0.51 and 0.47 L, respectively). This led to an estimated sample size of 380 subjects for T and 380 subjects for R to give 90% power to demonstrate clinical bioequivalence (T/R ratio and 90% confidence interval [CI] wholly contained within the 0.80–1.25 limits) between T and R, assuming a true T/R ratio of 1.0. The original sample size for the placebo group (*n* = 76) was based on performing a two-sided significance test at the 5% level with 99.9% power, an SD of 0.47 L, and a true mean difference from each active arm of 0.3 L for CFB in trough FEV_1_ on day 29 (allocation ratio of 5:1 for active to placebo). Therefore, the total number of subjects required to complete the study was 836 subjects (380 [T], 380 [R], and 76 [placebo]). This was rounded up to 935 subjects required to be randomized (425 [T], 425 [R], and 85 [placebo]) to allow for ∼10% dropout postrandomization. The process was repeated for the FEV_1_ AUC_(0–12)_ endpoint.

As sample size assumptions were based on historical reports of the effect of Advair Diskus in similar but not identical patient populations,^(19–21)^ a blinded sample size re-estimation (BSSR), which was prespecified in the protocol, was conducted for this study when 286 subjects had completed the study. The assumptions made about the CV for the original sample size calculation for CFB in trough FEV_1_ on day 29 were not supported by the aggregate data used for the BSSR. Therefore, the sample size was recalculated and revised accordingly. The total sample size to complete the study was revised from 836 to 990 subjects (450 [T] + 450 [R] + 90 [placebo]) requiring ∼1100 subjects to be randomized (the maximum allowable in the protocol). The revised sample size for the active treatment arms in this study was based on at least 81% power and assumed 112% between-subject CV (expected mean and SD for CFB in trough FEV_1_ on day 29 of 0.26 and 0.29 L, respectively, as assessed from the BSSR results). The sample size for the placebo group (*n* = 90) was chosen to maintain the allocation ratio (5:5:1).

Determination of assay sensitivity was required for the bioequivalence results to be valid. To evaluate assay sensitivity, comparisons of T versus placebo and R versus placebo were performed for day 1 FEV_1_ AUC_(0–12)_ and day 29 trough FEV_1_. A linear analysis of covariance (ANCOVA) model was fitted for each endpoint. Least-squares (LS) means were derived for each treatment, and LS mean differences were calculated for T versus placebo and for R versus placebo for each efficacy endpoint. Assay sensitivity was demonstrated if the *p*-values for all four comparisons (active treatment versus placebo for each FEV_1_ efficacy endpoint) were each less than 0.05.

To assess bioequivalence, LS means (one for T and one for R) from the ANCOVA models were used to generate T/R ratios for LS means for FEV_1_ AUC_(0–12)_ and trough FEV_1_ efficacy endpoints. Overall, 90% CIs were calculated by using Fieller's theorem.^(22)^ To demonstrate bioequivalence, the 90% CIs for the FEV_1_ AUC_(0–12)_ and trough FEV_1_ T/R ratios were each required to be wholly contained within the interval 0.80–1.25 (i.e., 80%–125%).

## Results

### Patients

**FIG. 1.**
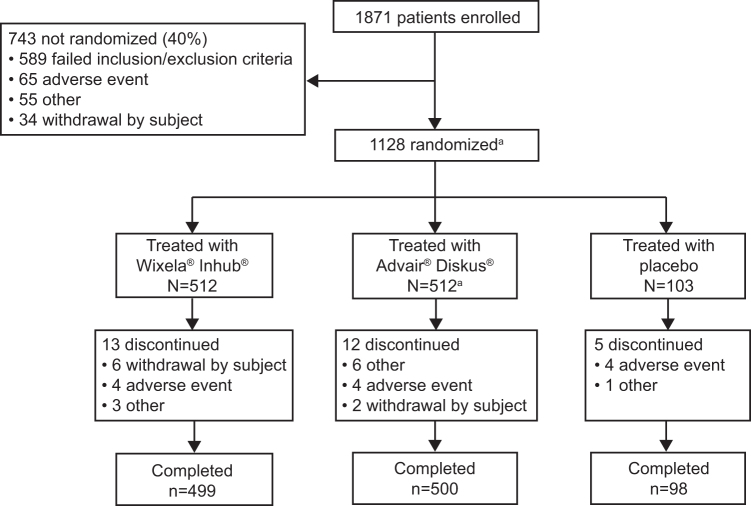
Patient flow. One of the patients randomized to the reference product (Advair Diskus) was not treated because of a failure to meet the inclusion/exclusion criteria, which resulted in 512 patients treated with Advair Diskus and 1127 total treated patients. All 1127 patients receiving a study treatment (Wixela Inhub [T], Advair Diskus [R], or placebo) were analyzed for safety. The FAS consisted of 1122 patients (509 [R], 511 [T], and 102 [placebo]); 5 treated patients (3 [T], 1 [R], and 1 [placebo]) were excluded from the FAS due to being enrolled into the study twice and so the second participations were excluded from the FAS. The PPS consisted of 1105 patients (502 [T and R], 101 [placebo]); 17 patients (7 [T], 9 [R], and 1 [placebo]) were excluded from the PPS due to ≥1 significant protocol deviation. FAS, full analysis set; PPS, per protocol set; R, reference product (Advair Diskus); T, test product (Wixela Inhub).

Of the 1871 enrolled patients, 1127 (60%) were randomized and treated, with 512 patients each receiving T and R and 103 patients receiving placebo (Fig. 1). The most common reason for exclusion of enrolled patients was failure to meet baseline spirometry criteria. All 1127 patients receiving a study treatment were analyzed in the safety set. The FAS consisted of 1122 patients (509 [R], 511 [T], and 102 [placebo]) whereas the PPS consisted of 1105 patients (502 [T], 502 [R], and 101 [placebo]). Of the randomized patients, 97% (*n* = 1097) completed the 4-week treatment period.

Baseline demographic and clinical characteristics were well matched across treatment groups (Table 1). In the total study population (safety set), mean age was 42.6 years, 40% of patients were male, mean duration (range) of asthma was 27.1 (0.3–79.8) years, mean (SD) FEV_1_ percent predicted was 69.94 (8.76), and mean percent improvement in FEV_1_ postbronchodilator was 23.84 (16.17). A total of 607 (54%) participants were taking ICS or ICS/LABA medication for maintenance of their asthma before entering the washout period of the study. Overall, 96% of patients in the safety set were compliant (i.e., within 75%–125% of per-protocol inhaler use) with treatment during the double-blind phase, and compliance was comparable for T (96%), R (95%), and placebo (97%).

**Table 1.** Baseline Demographic and Clinical Characteristics (Safety Set)

Characteristic	T (*n* = 512)	R (*n* = 512)	Placebo (*n* = 103)	Total (*n* = 1127)
Age, mean (range), years	42.6 (18–84)	42.5 (18–81)	43.5 (18–77)	42.6 (18–84)
Males, *n* (%)	206 (40.2)	203 (39.6)	39 (37.9)	448 (39.8)
Race, *n* (%)				
White	378 (73.8)	372 (72.7)	73 (70.9)	823 (73.0)
Black/African American	92 (18.0)	98 (19.1)	22 (21.4)	212 (18.8)
Other	42 (8.2)	42 (8.2)	8 (7.8)	92 (8.2)
BMI, mean (SD), kg/m^2^	29.4 (6.0)	29.1 (5.9)	29.4 (5.9)	29.3 (5.9)
Duration of asthma, mean (range), years	26.9 (0.3–79.8)	27.1 (0.8–70.7)	28.3 (0.6–65.8)	27.1 (0.3–79.8)
Prior asthma medication, *n* (%)				
ICS or ICS/LABA	275 (53.7)	272 (53.1)	60 (58.3)	607 (53.9)
ICS	86 (16.8)	97 (18.9)	20 (19.4)	203 (18.0)
ICS/LABA	189 (36.9)	175 (34.2)	40 (38.8)	404 (35.8)
Prebronchodilator spirometry				
*n*	512	511	103	1126
FEV_1_, mean (SD), L	2.33 (0.61)	2.32 (0.61)	2.28 (0.59)	2.32 (0.61)
FVC, mean (SD), L	3.46 (1.00)	3.41 (0.95)	3.42 (0.94)	3.43 (0.97)
FEV_1_/FVC, mean (SD), %	68.60 (9.05)	69.97 (9.30)	67.88 (9.39)	68.70 (9.19)
FEV_1_, mean (SD), % predicted	69.92 (8.64)	70.05 (8.83)	69.48 (9.03)	69.94 (8.76)
FEV_1_ reversibility				
*n*	511	511	103	1125
Improvement, mean (SD), %	23.23 (15.37)	24.43 (16.80)	23.97 (16.88)	23.84 (16.17)
Reversibility, mean (SD), L	0.53 (0.29)	0.55 (0.32)	0.53 (0.30)	0.54 (0.30)

BMI, body mass index; FEV_1_, forced expiratory volume in 1 second; FVC, forced vital capacity; ICS, inhaled corticosteroids; LABA, long-acting β-agonist; R, reference product (Advair Diskus); SD, standard deviation; T, test product (Wixela Inhub).

### Efficacy

**FIG. 2.**
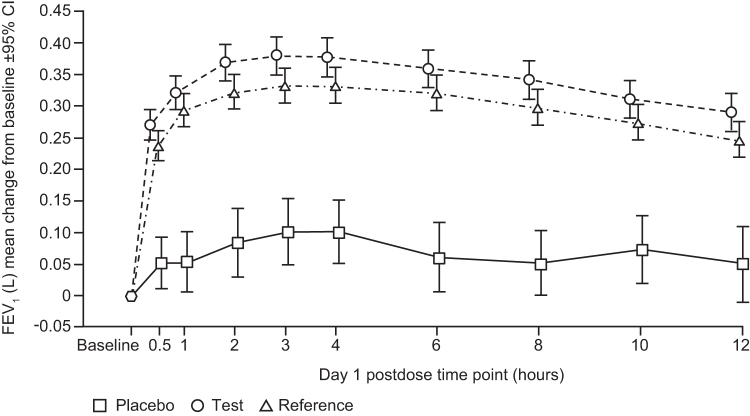
Change from baseline in FEV_1_ over time on day 1 with placebo (open squares), test (open circles), and reference (open triangles) FPS. Test FPS, Wixela Inhub; reference FPS, Advair Diskus. Data are mean and 95% CI. CI, confidence interval; FEV_1_, forced expiratory volume in 1 second. FPS, fluticasone propionate/salmeterol.

**FIG. 3.**
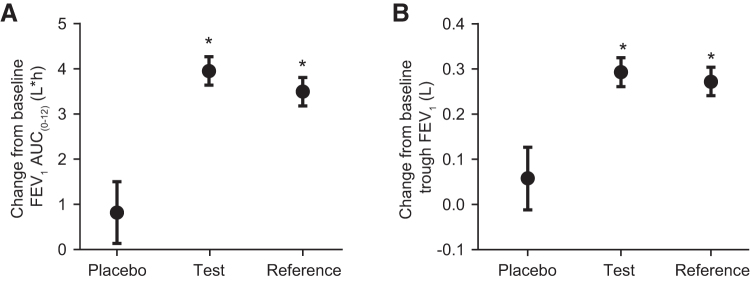
Day 1 **(A)** and day 29 **(B)** improvement in lung function after treatment with test and reference FPS and placebo. Baseline-subtracted data presented as LS mean and 95% CI. Test, Wixela Inhub; Reference, Advair Diskus. *Difference from placebo *p* < 0.0001. AUC_(0–12)_, area under the effect curve over 12 hours; LS, least squares.

Both active treatments substantially improved day 1 FEV_1_ by the first time point measured (mean CFB, T, 270 mL; R, 237 mL at 30 minutes postdose; Fig. 2); there was minimal improvement with placebo (mean CFB 52 mL). The maximum increase in FEV_1_ was observed at 3 hours postdose (mean CFB, T, 379 mL; R, 333 mL; placebo, 101 mL). T and R demonstrated similar FEV_1_ responses, with overlapping 95% CIs over the 12 hours of serial spirometry measures made on day 1 with clear separation from placebo (Fig. 2). LS mean increases in day 1 FEV_1_ AUC_(0–12)_ were comparable for T and R (3.953 and 3.496 L•h, respectively) and less for placebo 0.819 L•h (Fig. 3A and Table 2 [FAS]).

**Table 2.** Change from Baseline in FEV_1_ Auc_(0–12)_ on Day 1 and Trough FEV_1_ on Day 29 (FAS and PPS)

Treatment	FAS (primary analysis for assay sensitivity)			PPS (primary analysis for bioequivalence)		
	T	R	Placebo	T	R	Placebo
Day 1 change from baseline in FEV_1_ AUC_(0–12)_						
Model-adjusted FEV_1_ AUC_(0–12)_,^a^ L•h						
*n*	508	510	102	497	494	94
LS mean (SE)	3.953 (0.161)	3.496 (0.160)	0.819 (0.348)	3.973 (0.170)	3.541 (0.159)	0.840 (0.298)
95% CI	3.638 to 4.269	3.183 to 3.809	0.137 to 1.501	3.639 to 4.308	3.228 to 3.854	0.248 to 1.432
Difference from placebo^a^						
LS mean (SE)	3.134 (0.376)	2.677 (0.377)		3.133 (0.335)	2.701 (0.331)	
95% CI	2.396 to 3.872	1.938 to 3.417		2.472 to 3.795	2.047 to 3.3556	
*p*	<0.0001	<0.0001		<0.0001	<0.0001	
Equivalence test						
LS mean FEV_1_ T/R ratio	1.130			1.120		
90% CI	1.025 to 1.246			1.016 to 1.237		
Day 29 change from baseline in trough FEV_1_						
Model-adjusted change from baseline,^a^ L						
*n*	504	505	100	498	497	99
LS mean (SE)	0.293 (0.016)	0.272 (0.016)	0.058 (0.035)	0.291 (0.016)	0.273 (0.016)	0.057 (0.036)
95% CI	0.261 to 0.325	0.241 to 0.304	−0.012 to 0.127	0.259 to 0.323	0.241 to 0.305	−0.012 to 0.127
Difference from placebo^b^						
LS mean (SE)	0.235 (0.038)	0.215 (0.038)		0.234 (0.038)	0.215 (0.038)	
95% CI	0.161 to 0.310	0.140 to 0.289		0.159 to 0.309	0.140 to 0.291	
*p*	<0.0001	<0.0001		<0.0001	<0.0001	
Equivalence test						
LS mean trough FEV_1_ T/R ratio	1.079			1.069		
90% CI	0.948 to 1.230			0.938 to 1.220		

^a^ Based on ANCOVA.

^b^ Difference of LS means (T minus placebo, R minus placebo) from the ANCOVA model.

ANCOVA, analysis of covariance; AUC_(0–12)_, area under the effect curve over 12 hours postdose; CI, confidence interval; FAS, full analysis set; FEV_1_, forced expiratory volume in 1 second; LS, least squares; PPS, per protocol set; R, reference product (Advair Diskus); SE, standard error; T, test product (Wixela Inhub).

Both active treatments also substantially improved day 29 trough FEV_1_. The LS mean increases in CFB in trough FEV_1_ after twice-daily dosing for 28 days were 293 mL (T), 272 mL (R), and 58 mL (placebo) (Fig. 3B and Table 2 [FAS]).

LS mean increases over placebo in day 1 FEV_1_ AUC_(0–12)_ were 3.134 L•h (T) and 2.677 L•h (R), each *p* < 0.0001 versus placebo (Table 2 [FAS]), demonstrating clear clinical efficacy for the first dose of both active treatments. Both active treatments also significantly increased trough FEV_1_ over placebo after twice-daily dosing for 28 days with day 29 CFB in trough FEV_1_ of 235 mL [T] and 215 mL [R], each *p* < 0.0001 (Table 2 [FAS]).

As both T and R significantly increased day 1 FEV_1_ AUC_(0–12)_ and day 29 trough FEV_1_ over placebo (*p* < 0.0001; Table 2), the prespecified primary analysis criteria for assay sensitivity were met.

**FIG. 4.**
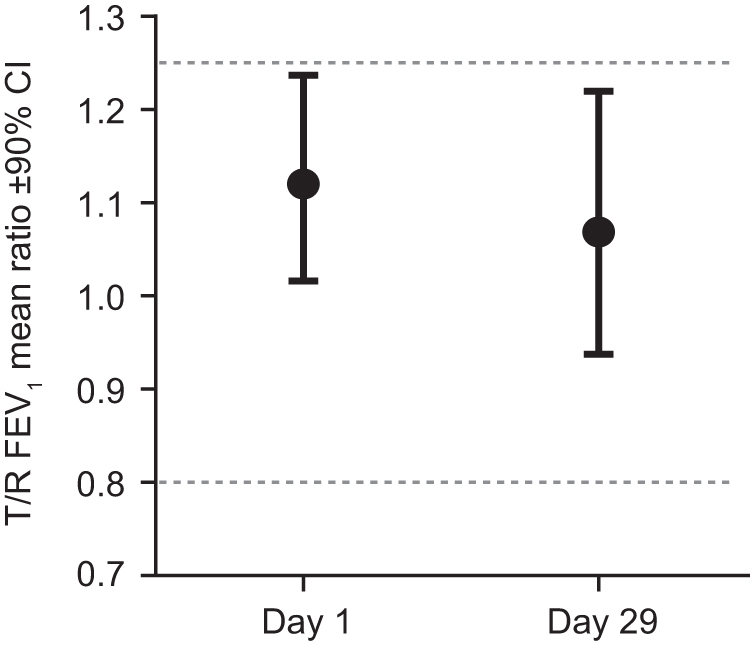
Day 1 and day 29 bioequivalence test. T/R FEV_1_ LS mean ratio and 90% CI for both day 1 (FEV_1_ AUC_(0–12)_) and day 29 (trough FEV_1_) co-primary endpoints were within the standard bioequivalence limits, shown as dotted lines. AUC_(0–12)_, area under the effect curve over 12 hours postdose; R, reference product (Advair Diskus); T, test product (Wixela Inhub).

Bioequivalence was then assessed, and the T/R ratios for LS means (90% CIs) for day 1 FEV_1_ AUC_(0–12)_ and day 29 trough FEV_1_ were 1.120 (1.016–1.237) and 1.069 (0.938–1.220), respectively (Table 2 [PPS]). As the 90% CIs for day 1 FEV_1_ AUC_(0–12)_ and day 29 CFB in trough FEV_1_ were between 0.80 and 1.25 (Fig. 4) for both endpoints, this indicated that T and R were bioequivalent on both endpoints.

### Safety

Treatment-emergent AEs occurred in 14.4% of patients in the safety set, with individual AEs displaying a similar incidence across the three treatment groups (Table 3). The percentage of asthma-related AEs was higher in the placebo group (4.9%) and lower and comparable in both active treatment groups (T, 1.4%; R, 2.0%). The percentage of discontinuations was also higher in the placebo group (4.9%) compared with the active treatment groups (T, 2.5%; R, 2.3%). The most commonly reported AEs were infections and respiratory disorders. No serious AEs or deaths occurred during the study period. AEs associated with FPS, such as oral candidiasis and dysphonia, occurred with a similarly low incidence in the T and R groups (candidiasis: 0.8% vs. 0.4%; dysphonia: 0.2% vs. 0.6%, respectively) and did not occur at all in the placebo group. A very low incidence (<1%) of AEs categorized as cardiac disorders was observed, all of which were mild in intensity, did not require intervention, and did not result in patients being withdrawn from the study. There were no clinically significant changes to laboratory safety tests, vital signs, or electrocardiograms.

**Table 3.** Treatment-Emergent Adverse Events by System Organ Class And Preferred Term (Safety Set)

Patients with AE,* n *(%)	T (*n* = 512)	R (*n* = 512)	Placebo (*n* = 103)
Any treatment-emergent AE	72 (14.1)	75 (14.6)	15 (14.6)
Infections and infestations	34 (6.6)	38 (7.4)	5 (4.9)
Upper respiratory tract infection	7 (1.4)	11 (2.1)	0
Nasopharyngitis	3 (0.6)	7 (1.4)	2 (1.9)
Respiratory, thoracic, and mediastinal disorders	15 (2.9)	25 (4.9)	7 (6.8)
Asthma	7 (1.4)	10 (2.0)	5 (4.9)
Oropharyngeal pain	3 (0.6)	5 (1.0)	1 (1.0)
Gastrointestinal disorders	6 (1.2)	5 (1.0)	1 (1.0)
Nervous system disorders	6 (1.2)	6 (1.2)	0
Headache	3 (0.6)	5 (1.0)	0
Musculoskeletal and connective tissue disorders	4 (0.8)	4 (0.8)	1 (1.0)
Injury, poisoning, and procedural complications	2 (0.4)	3 (0.6)	1 (1.0)
Cardiac disorders	3 (0.6)	0	1 (1.0)

Reported in ≥1% patients in the overall study population and/or ≥1% of any treatment group.

AE, adverse event; R, reference product (Advair Diskus); T, test product (Wixela Inhub).

## Discussion

Wixela Inhub was recently approved by the FDA as a fixed-dose FPS powder for oral inhalation to provide a generic equivalent to Advair Diskus. This study, recommended by the FDA for the clinical development of generic inhaled drugs containing FP and salmeterol powder,^(13)^ confirmed the local therapeutic equivalence of both the FP and salmeterol components of Wixela Inhub (T) after inhalation of 100/50 μg FPS, the lowest approved dose strength for Advair Diskus (R). Further, FPS administered as Wixela Inhub demonstrated a comparable safety profile to Advair Diskus.

A direct comparison of these results with those reported for the original Advair Diskus pivotal trials can be challenging, not only due to the expected limitations inherent in comparing between studies^(23)^ but also because of fundamental changes in the management of asthma itself over the past 20 years.^(24)^ As more asthma patients are treated with ICS and ICS/LABA therapy, the patients willing and able to participate in placebo-controlled studies may have a milder phenotype of asthma than those from 20 years ago and, hence, the opportunity to observe the magnitude of changes in lung function previously reported may be limited. A total of 54% of participants were taking ICS or ICS/LABA medication before the washout period in this study, of whom approximately one-third were taking an ICS without a LABA, and the remaining two-thirds were taking an ICS with a LABA. Thus, although the results (day 1 CFB in FEV_1_ AUC_(0–12)_: 3.95 L•h [T] and 3.50 L•h [R]; day 29 CFB in trough FEV_1_: 293 mL [T] and 272 mL [R]) are lower in absolute magnitude of lung function improvement than those originally reported for the same dose of Advair Diskus (5.81 L•h and 510 mL, respectively),^(19)^ the findings are otherwise consistent.

The day 1 and day 29 spirometry data were also comparable with those reported for the OT329 SOLIS bioequivalence study, which used an almost identical study design.^(7)^ For example, the day 1 CFB in FEV_1_ AUC_(0**–**12)_ for T (3.95 L•h) and R (3.50 L•h) were similar with respect to the magnitude of change with the corresponding day 1 values for OT329 SOLIS and Advair Diskus (3.72 and 3.55 L•h, respectively). In addition, the day 29 placebo-corrected CFB in trough FEV_1_ for T (235 mL) and R (215 mL) in this study are more similar to the corresponding day 29 values for OT329 SOLIS and Advair Diskus (168 and 163 mL, respectively) than to historical studies. The consistency of spirometry data across these more recently conducted studies suggests that the study design is robust, and the results are reproducible and representative of treatment effects in this population of asthma patients.

The design of this study was consistent with other FPS local therapeutic equivalence studies,^(7,9,21)^ and they adhered to the FDA guidelines for evaluation of local therapeutic equivalence for FPS products.^(13)^ The use of the lowest of three dose strengths of the FP component approved for Advair Diskus was appropriate, because it was the most likely to identify any treatment differences in FP between T and R and consistent with the FDA guidance. The use of higher dose strengths, which elicit maximal responses of FPS in many patients,^(25)^ might have masked potential differences between T and R and resulted in erroneous conclusions. We acknowledge, however, that international regulatory agencies may have different requirements for study designs and doses to be studied for the demonstration of local therapeutic equivalence.^(26)^

Due to the difference in physical appearance of T and R, each treatment was administered twice daily for 28 days in a double-blind manner, using the double-dummy technique^(27)^ with placebo inhalers matched to T or R. This can be considered a gold standard for clinical trials and contrasts with the bioequivalence study for OT329 SOLIS, in which the placebo treatment group received the placebo for the SOLIS inhaler only and hence the T and R were not blinded between each other.^(7)^ Use of a double-dummy technique, which increases the robustness of conclusions of randomized trials of experimental interventions,^(28)^ is a strength of this study. This robust double-dummy study design was also employed in local therapeutic equivalence trials of a novel dry powder FPS inhaler (AirFluSal^®^ Forspiro^®^; Sandoz International GmbH, Holzkirchen, Germany)^(9)^ and a chlorofluorocarbon-free metered-dose FPS inhaler.^(21)^

The results of the primary analyses were corroborated by secondary analyses (assay sensitivity [PPS] and bioequivalence [FAS]) that showed that (1) day 1 FEV_1_ AUC_(0–12)_ and day 29 trough FEV_1_ endpoints were significantly superior to placebo for both T and R (*p* < 0.0001) and (2) T was bioequivalent to R for both co-primary endpoints.

The demonstration of local therapeutic equivalence using spirometry endpoints in this article is also supported by previously presented data on pharmacokinetic bioequivalence to all three-dose strengths of Advair Diskus (100/50, 250/50, and 500/50 μg FP/S) (Haughie et al., 2019), *in vitro* equivalence (e.g., emitted dose) at all three dose strengths,^(18)^ as well as meeting all of the FDA requirements for device equivalence.^(17)^

In conclusion, Wixela Inhub, which was recently approved by the FDA, will represent a new generic-equivalent FPS treatment option for asthmatic patients whose symptoms are uncontrolled with ICS alone and COPD patients at high risk of exacerbations.

## Notes

Reviewed by:

Dennis Sandell

Peter Fan

## References

[1] Yawn BP, Raphiou I, Hurley JS, and Dalal AA: The role of fluticasone propionate/salmeterol combination therapy in preventing exacerbations of COPD. Int J Chron Obstruct Pulmon Dis. 2010;5:165–178 20631816 10.2147/copd.s4159 PMC2898089

[2] McKeage K, and Keam SJ: Salmeterol/fluticasone propionate: A review of its use in asthma. Drugs. 2009;69:1799–1828 19719334 10.2165/11202210-000000000-00000

[3] NAEPP: Expert panel report 3: Guidelines for the diagnosis and management of asthma. 2007. https://www.nhlbi.nih.gov/files/docs/guidelines/asthgdln.pdf (accessed 1016, 2018) 10.1016/j.jaci.2007.09.043 17983880

[4] GINA: GINA report Global Strategy for Asthma Management and Prevention 2018. https://ginasthma.org/2018-gina-report-global-strategy-for-asthma-management-and-prevention (accessed 1016, 2018)

[5] GOLD. Global Initiative for Chronic Obstructive Lung Disease (GOLD). 2017. http://bit.ly/2hNAJ9K (accessed 213, 2017)

[6] Steinfeld J, Yiu G, and Miller SD: Dose-ranging study to evaluate the efficacy and safety of four doses of fluticasone propionate/salmeterol multidose dry powder inhaler (FS MDPI) compared with fluticasone propionate (FP) MDPI and FS DPI in subjects with persistent asthma. J Allergy Clin Immunol. 2015;135:AB6 [Abstract].

[7] Longphre MV, Getz EB, and Fuller R: Clinical bioequivalence of OT329 SOLIS and ADVAIR DISKUS in adults with asthma. Ann Am Thorac Soc. 2017;14:182–189 27849125 10.1513/AnnalsATS.201606-436OC

[8] Gerald JK: Generic competition for orally inhaled respiratory medications. Two steps forward, one step back. Ann Am Thorac Soc. 2017;14:165–167 28146386 10.1513/AnnalsATS.201611-899ED

[9] Kuna P, Thyroff-Friesinger U, Gath I, and Jones S: Randomized equivalence trial: A novel multidose dry powder inhaler and originator device in adult and adolescent asthma. Allergy Asthma Proc. 2015;36:352–364 26219538 10.2500/aap.2015.36.3886

[10] US National Library of Medicine: An open study to assess the robustness of the CRC749 inhaler. 2015. https://clinicaltrials.gov/ct2/show/NCT02474017 (accessed 82, 2019)

[11] US National Library of Medicine: Advair Diskus local equivalence study in asthma. 2014. https://clinicaltrials.gov/ct2/show/NCT02245672 (accessed 82, 2019)

[12] Mylan. Mylan announces FDA approval of Wixela™ Inhub™ (fluticasone propionate and salmeterol inhalation powder USP), first generic of ADVAIR DISKUS^®^ (fluticasone propionate and salmeterol inhalation powder). 2019. http:/newsroom.mylan.com/2019-01-31-Mylan-Announces-FDA-Approval-of-Wixela-TM-Inhub-TM-fluticasone-propionate-and-salmeterol-inhalation-powder-USP-First-Generic-of-ADVAIR-DISKUS-R-fluticasone-propionate-and-salmeterol-inhalation-powder (accessed 82, 2019)

[13] Food and Drug Administration: Draft guidance on fluticasone propionate; salmeterol xinafoate. 2013. https://www.fda.gov/downloads/drugs/guidancecomplianceregulatoryinformation/guidances/ucm367643.pdf (accessed 1016, 2018)

[14] Haughie S, Allan R, Wood N, and Ward J: Equivalent systemic exposure to fluticasone propionate/salmeterol following single inhaled doses from Advair^®^ Diskus^®^ and Wixela^®^ Inhub^®^: Results of three pharmacokinetic bioequivalence studies. J Aerosol Med Pulm Drug Deliv. 2019 [Epub ahead of print]; DOI: 10.1089/jamp.2019.1537 PMC7041328 31364911

[15] International Council for Harmonisation: International Council for Harmonisation of Technical Requirements for Pharmaceuticals for Human Use: Integrated addendum to ICH harmonised guideline: Guideline for good clinical practice E6 (R2); 2016

[16] World Medical Association: WMA Declaration of Helsinki—Ethical principles for medical research involving human subjects. 2018. https://www.wma.net/policies-post/wma-declaration-of-helsinki-ethical-principles-for-medical-research-involving-human-subjects (accessed 1016, 2018)

[17] Allan R, Newcomb C, Canham K, Wallace R, and Ward J: Usability and robustness of the Wixela^®^ Inhub^®^ dry powder inhaler. Am J Respir Crit Care Med. 2019;199:A2206 10.1089/jamp.2020.1603 PMC8060712 32865454

[18] Cooper A, Newcomb C, Wallace R, Canham K, Ward J, Allan R, Berry M, Parker J, and Clift E: Wixela^®^ Inhub^®^ dry powder inhaler—In vitro performance compared with advair diskus and inhalation profiles in patients with asthma or chronic obstructive pulmonary disease. Am J Respir Crit Care Med. 2019;199:A2207

[19] Kavuru M, Melamed J, Gross G, Laforce C, House K, Prillaman B, Baitinger L, Woodring A, and Shah T: Salmeterol and fluticasone propionate combined in a new powder inhalation device for the treatment of asthma: A randomized, double-blind, placebo-controlled trial. J Allergy Clin Immunol. 2000;105:1108–1116 10856143 10.1067/mai.2000.105711

[20] Shapiro G, Lumry W, Wolfe J, Given J, White MV, Woodring A, Baitinger L, House K, Prillaman B, and Shah T: Combined salmeterol 50 microg and fluticasone propionate 250 microg in the diskus device for the treatment of asthma. Am J Respir Crit Care Med. 2000;161:527–534 10673196 10.1164/ajrccm.161.2.9905091

[21] Bateman ED, Silins V, and Bogolubov M: Clinical equivalence of salmeterol/fluticasone propionate in combination (50/100 microg twice daily) when administered via a chlorofluorocarbon-free metered dose inhaler or dry powder inhaler to patients with mild-to-moderate asthma. Respir Med. 2001;95:136–146 11217910 10.1053/rmed.2000.1008

[22] Fieller EC: Some problems in interval estimation. J R Stat Soc Series B Stat Methodol. 1954:175–185

[23] Pocock SJ, Hughes MD, and Lee RJ: Statistical problems in the reporting of clinical trials. N Engl J Med. 1987;317:426–432 3614286 10.1056/NEJM198708133170706

[24] Boulet LP, FitzGerald JM, and Reddel HK: The revised 2014 GINA strategy report: Opportunities for change. Curr Opin Pulm Med. 2015;21:1–7 25405667 10.1097/MCP.0000000000000125

[25] GlaxoSmithKline: ADVAIR DISKUS prescribing information. 2017. https://www.accessdata.fda.gov/drugsatfda_docs/label/2017/021077s056s057lbl.pdf (accessed 1016, 2018)

[26] Lu D, Lee SL, Lionberger RA, Choi S, Adams W, Caramenico HN, Chowdhury BA, Conner DP, Katial R, Limb S, Peters JR, Yu L, Seymour S, and Li BV: International guidelines for bioequivalence of locally acting orally inhaled drug products: Similarities and differences. AAPS J. 2015;17:546–557 25758352 10.1208/s12248-015-9733-9 PMC4406956

[27] Day SJ, and Altman DG: Statistics notes: Blinding in clinical trials and other studies. Br Med J. 2000;321:19–26 10.1136/bmj.321.7259.504 PMC1118396 10948038

[28] Pildal J, Hrobjartsson A, Jorgensen KJ, Hilden J, Altman DG, and Gotzsche PC: Impact of allocation concealment on conclusions drawn from meta-analyses of randomized trials. Int J Epidemiol. 2007;36:847–857 17517809 10.1093/ije/dym087

